# PD-1 inhibitor combined with TPF induction chemotherapy in locally advanced nasopharyngeal carcinoma: a retrospective study of efficacy and safety

**DOI:** 10.3389/fimmu.2025.1654616

**Published:** 2025-09-16

**Authors:** Weiwei Zhang, Yousheng Meng, Ping Zhang, Dujuan Tian, Xianghua Zeng, Mingqing Dong, Lang He

**Affiliations:** ^1^ Cancer prevention and treatment institute of Chengdu, Department of oncology, Chengdu Fifth People’s Hospital/The Second Clinical Medical College, Affiliated Fifth People’s Hospital of Chengdu University of Traditional Chinese Medicine, Chengdu, China; ^2^ Division of Pulmonary Medicine, the First Affiliated Hospital, Wenzhou Medical University, Wenzhou Key Laboratory of Interdiscipline and Translational Medicine, Wenzhou Key Laboratory of Heart and Lung, Wenzhou, China

**Keywords:** locally advanced stage nasopharyngeal, TPF, PD-1 inhibitor, concurrent chemoradiotherapy, efficacy and safety

## Abstract

**Background:**

The efficacy of PD-1 inhibitors in the induction therapy of locally advanced nasopharyngeal carcinoma (LA-NPC) remains unclear. The aim of this study was to retrospectively investigate the efficacy and safety of PD-1 inhibitor combined with induction chemotherapy in patients with LA-NPC.

**Patients and methods:**

A retrospective study was conducted on 158 LA-NPC patients, 80 patients received TPF (nab-paclitaxel, cisplatin and 5-fuorouracil) induction chemotherapy, and 78 patients received TPF-ICB (TPF plus PD-1 inhibitor) chemoimmunotherapy. Treatment response was evaluated immediately following completion of induction therapy using RECIST v1.1 criteria, including cervical lymph nodes and primary nasopharynx lesions. Responses were categorized as complete response (CR), partial response (PR), stable disease (SD), or progressive disease (PD), with objective response rate (ORR) calculated as the combined CR+PR rate. Secondary endpoints included progression-free survival (PFS), overall survival (OS), and toxicity assessment. Acute treatment-related toxicities during induction therapy were graded according to CTCAE v5.0 criteria and compared between treatment groups.

**Results:**

After induction therapy, the ORR in the TPF group was significantly lower than that in the TPF-ICB group (71.2% vs. 88.5%, *p* = 0.007). The complete response (CR) rate in the TPF-ICB group was significantly higher than in the TPF group (29.5% vs. 11.3%, *p* = 0.004). The 3 and 5 years PFS rates in TPF-ICB group were 99% and 95%, which were significantly higher than the TPF group (89% and 87%, both *p* < 0.05). The 3-year (99% vs. 89%, *p* <0.001) and 5-years OS rates (95% vs. 87%, *p* < 0.0001) were superior in the TPF-ICB group. Grade ≥3 TRAEs occurred in 7 (8.6%) and 12 (15.5%) patients in the TPF and TPF-ICB groups, respectively (*p* = 0.596).

**Conclusions:**

The induction therapy of PD-1 inhibitor combined with TPF showed high CR and ORR rates in LA-NPC, and the safety was acceptable.

## Introduction

Nasopharyngeal carcinoma (NPC) is a distinct subtype of head and neck cancer with a unique geographical distribution, showing high prevalence in endemic regions such as Southeast Asia and Southern China (1–3). Locally advanced stages (Stage III-IVA) account for approximately 70% of newly diagnosed cases, presenting significant therapeutic challenges due to their propensity for local invasion and distant metastasis (4, 5). Significant therapeutic advances have been achieved in locally advanced nasopharyngeal carcinoma (LA-NPC), where meta-analyses have established concurrent chemoradiotherapy (CCRT) as superior to radiotherapy alone in improving survival outcomes (6). Modern radiation strategies employing dose escalation (primary tumor dose range: 68.1-74.25 Gy, median 70.4 Gy) with selective boost irradiation (median 9 Gy) demonstrate improved prognosis, particularly for T3-T4 disease (7). However, despite these advances including intensity-modulated radiotherapy (IMRT), 20-30% of patients still experience treatment failure, predominantly due to distant metastasis, highlighting the urgent need for more effective systemic strategies and optimized integration of induction chemotherapy (6, 8–15).

Induction chemotherapy (IC) has emerged as a promising approach to mitigate the risk of micrometastases and reduce tumor burden prior to definitive radiotherapy (16). Several phase III trials, including the NEOC-002 and GP-2018 studies, have demonstrated that IC with gemcitabine and cisplatin (GP) or docetaxel, cisplatin, and 5-fluorouracil significantly improves progression-free survival (PFS) and distant metastasis-free survival (DMFS) compared to CCRT alone (8, 17). However, the optimal integration of IC with novel immunotherapeutic agents, particularly programmed death-1 (PD-1) inhibitors, remains an area of active investigation. Immune checkpoint inhibitors (ICIs) have revolutionized cancer treatment by reinvigorating antitumor immunity, and their role in NPC is supported by the remarkable efficacy observed in recurrent or metastatic settings (18). Given the robust expression of PD-L1 in EBV-associated NPC and the immunogenic nature of the disease, combining IC with PD-1 blockade may further enhance treatment outcomes by priming the tumor microenvironment for immune-mediated cytotoxicity (19).

In recent years, breakthrough progress has been made in the blockade therapy of suppressive immune checkpoint PD-1/programmed death ligand-1 (PD-L1) in NPC. The effectiveness and safety of many PD-1/PD-L1 inhibitors have been demonstrated in patients with recurrent or metastatic NPC (R/M-NPC) (20, 21). Many studies have confirmed the safety and efficacy of PD-1 inhibitors combined with chemotherapy in first-line treatment of R/M-NPC patients (20, 22–25). In addition, based on the results of three large-scale clinical studies, NCT03121716, NCT03581786, CAPTAIN-1<sup>st</sup> and RATIONALE-309, camrelizumab, tislelizumab, toripalimab and sintilimab combined with gemcitabine and cisplatin (GP) regimen have been approved in China for first-line standard treatment of R/M-NPC (20–23, 26).

Clinical evidence from immune checkpoint inhibitors in recurrent/metastatic NPC (R/M-NPC) suggests that IC combined with immunotherapy may be a promising treatment option for LA-NPC (20, 22, 23). A randomized phase III trial in LA-NPC demonstrated that patients receiving sintilimab plus standard GP regimen induction chemotherapy and concurrent chemoradiotherapy achieved a significantly higher 3-year event-free survival rate of 86.1%, compared to 76% in the standard treatment group (*p* = 0.019) (27). These findings support the synergistic antitumor effect of immunotherapy combined with chemotherapy in NPC and highlight its potential in LA-NPC treatment. However, the efficacy and safety of anti-PD-1 therapy in LA-NPC remain uncertain and require further real-world validation. While existing studies confirm the benefit of PD-1 inhibitors with GP-based induction chemotherapy, their synergy with TPF (nab-paclitaxel, cisplatin, and 5-fluorouracil) induction remains unclear.

Despite the biological rationale, clinical data on combining IC with PD-1 inhibitors in LA-NPC remain limited. Preliminary evidence from the phase II CAPTAIN study demonstrated that camrelizumab monotherapy achieved a 28.2% objective response rate (ORR) in heavily pretreated recurrent/metastatic NPC, with biomarker analysis suggesting MHC cell density and PD-L1 expression may predict response (23). In contrast, the phase III JUPITER-02 trial established toripalimab combined with gemcitabine-cisplatin as a new first-line standard for recurrent/metastatic NPC, showing significant improvements in both progression-free survival (median 21.4 vs 8.2 months) and overall survival (HR 0.63) compared to chemotherapy alone (26). However, most studies focus on metastatic disease, leaving a gap in evidence for non-metastatic LA-NPC. Additionally, long-term survival benefits, toxicity profiles, and predictive biomarkers for this combination need further investigation. This retrospective study aims to evaluate the efficacy and safety of TPF chemotherapy (nab-paclitaxel, cisplatin and 5-fuorouracil) plus PD-1 inhibitors (TPF-ICB) in LA-NPC, providing real-world evidence to guide clinical practice. By analyzing survival outcomes and treatment-related adverse events, we aim to contribute to the evolving role of immunotherapy in NPC management.

## Methods

### Ethics consideration

The retrospective study was approved by the ethics Committee of Chengdu Fifth People’s Hospital/The Second Clinical Medical College, Affiliated Fifth People’s Hospital of Chengdu University of Traditional Chinese Medicine Committee. All participants were asked to sign an informed consent form. All methods were performed in accordance with the relevant guidelines and regulations and adhered to the ethical standards of the institutional and national research committee as well as with the 1964 Helsinki Declaration (along with its later amendments or similar ethical standards).

### Patient selection

This study aims to retrospectively investigate the efficacy and safety of PD-1 inhibitors combined with TPF induction chemotherapy in LA-NPC patients. Patients were enrolled if they met these criteria: with untreated locally advanced III-IVA stage NPC based on the 8th AJCC classification; an Eastern Cooperative Oncology Group (ECOG) performance status of 0 – 1; and adequate marrow and organ function. The main exclusion criteria included the combination of a second malignancy; a previous or ongoing active autoimmune disease; acquired Immune Deficiency Syndrome (AIDS); active hepatitis B virus (HBV) or hepatitis C virus (HCV) infection and active tuberculosis; and incomplete follow-up data.

### Induction chemotherapy and immunotherapy

Between January 2018 and December 2023, 158 patients with untreated III-IVA stage LA-NPC (cT1-2 N2-3 or cT3-4 N0-3 based on the American Joint Committee on Cancer (AJCCC) 8<sup>th</sup> edition) met the inclusion and exclusion criteria of this study. All patients received induction TPF chemotherapy every 3 weeks for three cycles: nab-paclitaxel (Shiyao Group Ouyi, Shijiazhuang, China) 260 mg/m^2^ on day 1, cisplatin (Haosen, Jiangsu, China) 75 mg/m^2^ on day 1, and 5-fluorouracil (Xudonghaipu, Shanghai, China) 600 mg/m^2^ per day, continuous intravenous infusion day 1-5 (16, 28, 29). Among them, 78 patients received TPF-ICB treatment, including sintilimab (Innovent Biologics, Suzhou, China), toripalimab (Zhonghe Biomedical, Suzhou, China) or tislelizumab (BeiGene, Guangzhou, China). All PD-1 inhibitors are administered intravenously at a dose of 200 mg on the first day, every 3 weeks. All chemotherapy drugs were sourced from the hospital pharmacy, compliant with National Medical Products Administration (NMPA) regulations, and prepared under standardized protocols. Dose modifications followed guideline-based criteria.

### Concurrent chemoradiotherapy

All patients received 3 cycles of IC followed by CCRT, with radiotherapy administered using intensity-modulated radiotherapy (IMRT) techniques according to NPC treatment guidelines (16). During CCRT, weekly cisplatin (40 mg/m²) was delivered with a planned cumulative dose ≥200 mg/m² (30). The delineation of IMRT target area is determined according to the guidelines of NPC experts (3, 31).

The IMRT target volumes included the primary gross tumor volume (GTVp, 70.95 Gy/33 fractions), metastatic lymph nodes (GTVn, 66 Gy/33 fractions), high-risk clinical target volume (CTV1, 60 Gy/30 fractions), and low-risk clinical target volume (CTV2, 54 Gy/30 fractions), with treatments delivered five times weekly, consistent with established protocols (28, 32, 33).

### Study endpoints

The main endpoint of this study is the clinical ORR after 3 cycles of induction therapy, and it is required to be done 1 week before the start of radiotherapy. Tumor response assessment was performed 1 week following induction therapy completion through: (1) systematic nasopharyngoscopic evaluation and (2) contrast-enhanced magnetic resonance imaging (MRI) of the nasopharynx and neck (or computed tomography (CT) for patients with MRI contraindications). Secondary endpoints included PFS, overall survival (OS), and toxicity assessment. According to the Response Evaluation Criteria in Solid Tumors (RECIST version 1.0) (34, 35), response was categorized as complete response (CR), partial response (PR), stable disease (SD), and progression of disease (PD). ORR refers to the number of cases achieving CR or PR as a proportion of the number of evaluable cases. Toxicity during induction chemotherapy or immunotherapy was graded according to the National Cancer Institute Common Terminology Criteria for Adverse Events (version 5.0).

### Survival and prognostic factors

PFS was defined as the time from the start of the patient’s induction chemotherapy with or without immunotherapy to PD or death from any cause. OS was calculated from the time of initial diagnosis to the time of death from any cause. Kaplan Meier survival curve plot describes the PFS and OS of patients. Cox univariate and multivariate analyses were used to determine prognostic indicators for PFS and OS in patients with NPC.

### Follow-up

Follow-up procedures were systematically conducted at 3-month intervals in years 0 – 3, at 6-month intervals in years 3 – 5, and annually thereafter (until death). The comprehensive evaluations comprising: (1) physical examinations with nasopharyngeal endoscopy; (2) head and neck MRI/CT; (3) serum tumor marker assessment; (4) quantitative Epstein-Barr virus DNA monitoring. The final follow-up data were updated on June 1, 2024. For survival analysis, all time-to-event outcomes were calculated from the initial diagnosis date until either the occurrence of the defined event or the final follow-up contact.

### Statistical analysis

Continuous variables were analyzed using Student’s T-tests; categorical variables were analyzed using chi-square tests. The chi-square test was used to compare clinical pathologic variables, therapeutic efficacy, ORR, and toxicity between the two groups. The cutoff values of age and the Ki67 index were calculated using ROC curves. The Kaplan–Meier method was used to compare the PFS and OS between the two groups. A Cox model was subsequently used to assess the independence of immunotherapy in affecting the PFS and OS. All statistical analyses were performed using SPSS 25.0. The differences of *p* < 0.05 were considered statistically significant.

## Results

### Patient characteristics

This study included 158 LA-NPC patients meeting the inclusion criteria, comprising 80 patients receiving TPF chemotherapy alone and 78 receiving PD-1 inhibitors plus TPF. The immunotherapy subgroup (n=80) included sintilimab (33 patients), tislelizumab (27), and toripalimab (20). The cohort consisted of 108 males (68.4%) and 50 females (31.6%), with a mean age of 55.4 years. Tumor staging distribution was: T1 (5.1%), T2 (38.0%), T3 (37.3%), and T4 (19.6%); nodal staging was N0 (2.5%), N1 (14.6%), N2 (60.8%), and N3 (22.2%). Disease stages were III (58.2%) and IV (41.8%).

Demographics showed 24.7% patients <50 years and 75.3% ≥50 years, with 63.3% non-smokers and 83.3% non-drinkers. ECOG performance status was 0 (42.4%) or 1 (57.6%). Based on Ki67 proliferation index (low: <10%; moderate: 10-30%; high: ≥30%), patients were stratified into high-proliferation (Ki67≥30%) and intermediate/low-proliferation (Ki67<30%) groups. Body mass index (BMI) was calculated as weight (kg)/height² (m²). The groups showed comparable distributions, with no significant differences found in baseline characteristics, including age, gender, ECOG status, disease stage, Ki67, BMI, smoking, and drinking status (**Table 1**; all *p* > 0.05).

**Table 1:** Baseline clinical characteristics of patients with LA-NPC in different treatment groups.

Variables	TPF–ICB (n=78)	TPF (n=80)	Total (n=158)	*P* value
Age (years)				0.644
≤50	18(23.1%)	21(26.2%)	39(24.7%)	
>50	60(76.9%)	59(73.8%)	119(75.3%)	
Gender				0.208
Male	57(73.1%)	51(63.8%)	108(68.4%)	
Female	21(26.9%)	29(36.2%)	50(31.6%)	
Smoker				0.59
Yes	27(34.6%)	31(38.8%)	58(36.7%)	
No	51(65.4%)	49(61.2%)	100(63.3%)	
Drinker				0.354
Yes	8(10.3%)	12(15.2%)	20(12.7%)	
No	70(89.7%)	67(84.8%)	137(87.3%)	
Ki67				0.118
≤30%	22(28.2%)	32(40.0%)	54(34.2%)	
>30%	56(71.8%)	48(60.0%)	104(65.8%)	
ECOG score				0.536
0	35(44.9%)	32(40.0%)	67(42.4%)	
1	43(55.1%)	48(60.0%)	91(57.6%)	
Disease stage				0.61
III	47(60.3%)	45(56.2%)	92(58.2%)	
IVA	31(39.7%)	35(43.8%)	66(41.8%)	
Tumor stage				0.984
T1	4(5.1%)	4(5.0%)	8(5.1%)	
T2	30(38.5%)	30(37.5%)	60(38.0%)	
T3	28(35.9%)	31(38.8%)	59(37.3%)	
T4	16(20.5%)	15(18.8%)	31(19.6%)	
Neck stage				0.739
N0	2(2.6%)	2(2.5%)	4(2.5%)	
N1	9(11.5%)	14(17.5%)	23(14.6%)	
N2	50(64.1%)	46(57.5%)	96(60.8%)	
N3	17(21.8%)	18(22.5%)	35(22.2%)	
BMI (kg/m<sup>2</sup>)				0.18
≤18.5	4(5.1%)	11(13.8%)	15(9.5%)	
18.5-24	54(69.2%)	51(63.8%)	105(66.5%)	
≥24	20(25.6%)	18(22.5%)	38(24.1%)	
CR				0.004
Yes	23(29.5%)	9(11.3%)	32(20.3%)	
No	55(70.5%)	71(88.9%)	126(79.7%)	

BMI, Body mass index; CR, Complete response; ECOG, Eastern Cooperative Oncology Group; ICB, PD-1 inhibitor immunotherapy; N, lymph node; T, tumor; TPF, nab-paclitaxel, cisplatin, and 5-fluorouracil.

### Treatment response

After induction therapy, the CR, PR, and SD of TPF group occurred in 9 (11.3%), 48 (60.0%), and 23 (28.7%) patients, respectively. In the TPF-ICB group, CR, PR, and SD occurred in 23 (29.5%), 46 (59.0%), and 9 (11.5%) patients, respectively. The ORR in the TPF-ICB group was significantly higher than that in the TPF group (88.5% vs. 71.2%, *p* = 0.007) (**Table 2**).

**Table 2:** Results of tumor efficacy evaluation.

Therapeutic effect	TPF–ICB group (n=78)	TPF group (n=80)	*P* value
Complete response	23(29.5%)	9(11.3%)	0.004
Partial response	46(59.0%)	48(60.0%)	0.0896
Stable disease	9(11.5%)	23(28.7%)	0.007
Objective remission rate	88.50%	71.20%	0.007
2-year PFS rate	99%	89.00%	0.018
3-year PFS rate	97%	84%	<0.0001
5-year PFS rate	95.00%	84.00%	<0.0001
2-year OS rate	99%	92.00%	0.102
3-year OS rate	99%	89%	<0.001
5-year OS rate	95.00%	87.00%	<0.0001

ICB, PD-1 inhibitor immunotherapy; OS, overall survival; PFS, progression free survival; TPF, nab-paclitaxel, cisplatin, and 5-fluorouracil.

The median follow-up time was 65 months (range: 10 – 78 months). Due to the median PFS and OS not being reached, 2, 3 and 5 years PFS and OS rate were reported. The 2-year PFS rate of patients in the TPF-ICB group was 99%, significantly higher than that in the TPF group (89%, *p* = 0.018). The 3-year and 5-year PFS rates of patients in the TPF-ICB group were 97% and 95%, respectively, both significantly higher than the 84% (*p* < 0.0001) and 84% (*p* < 0.0001) rates in the TPF group (**Table 2**).

The 2-year OS rate of patients in the TPF-ICB group was 99%, with no statistically significant difference compared to the TPF group (92%, *p* = 0.102). The 3-year and 5-year OS rates of patients in the TPF-ICB group were 99% and 95%, respectively, both significantly higher than the 89% (*p* <0.001) and 87% (*p* <0.0001) rates in the TPF group (**Table 2**).

**Figure 1:**
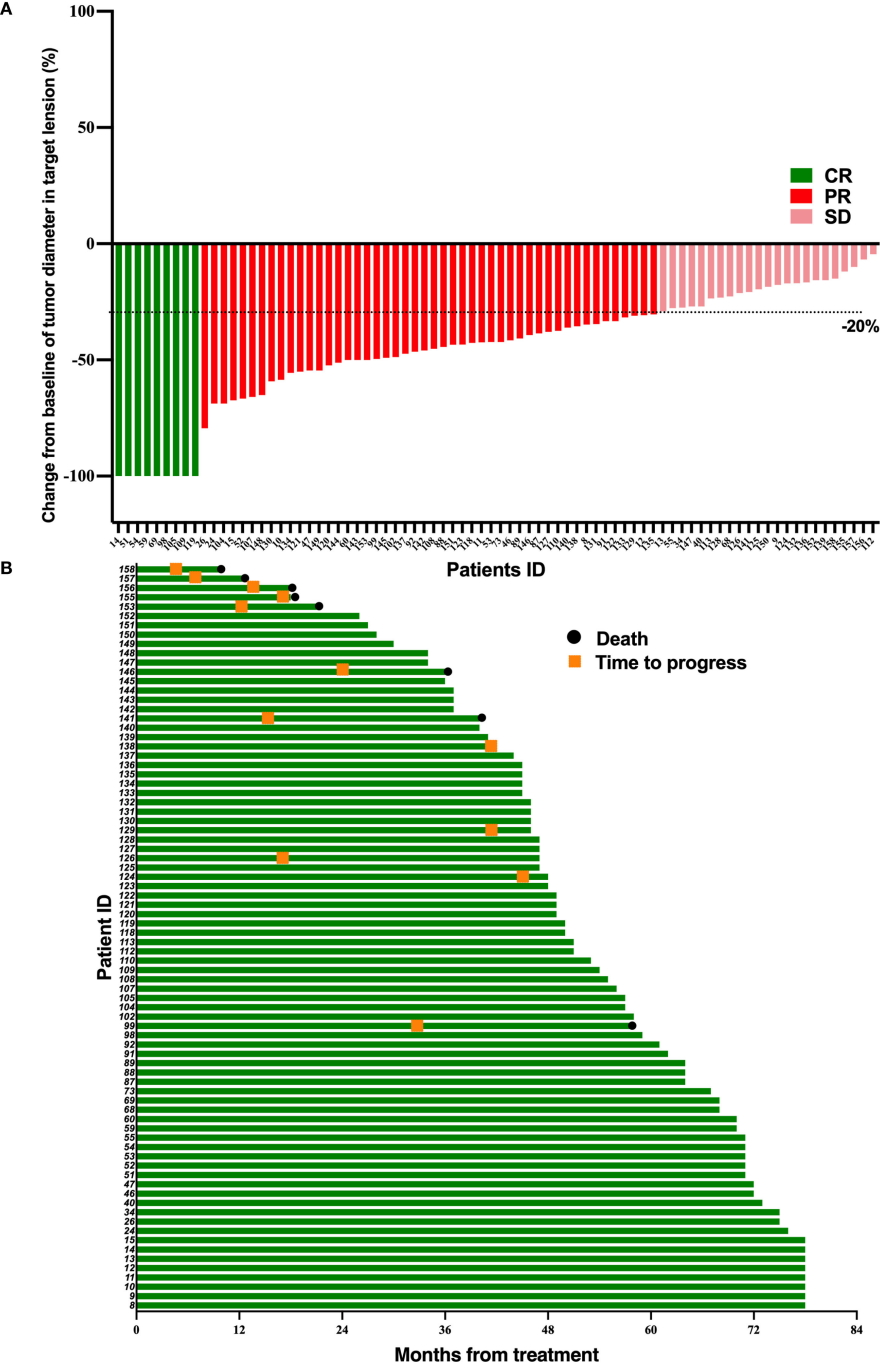
Tumor response and duration of response in 80 LA-NPC patients receiving simple induction chemotherapy. **(A)** Percentage reduction of tumor diameter of the primary lesion compared with baseline with response evaluation criteria in solid tumors version 1.1 (RECIST v1.1) measurement. **(B)** The duration of response in patients undergoing induction chemotherapy.

**Figure 2:**
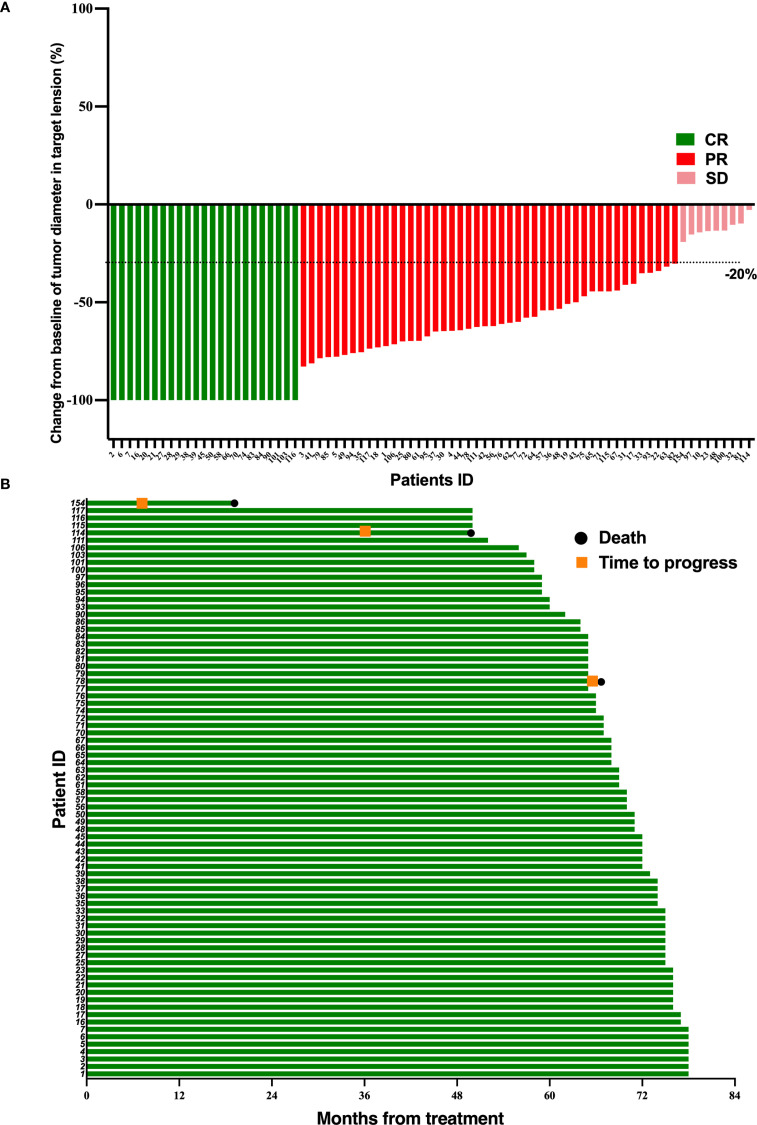
Tumor response and duration of response in 78 LA-NPC patients receiving PD-1 inhibitor and chemotherapy. **(A)** Percentage reduction of tumor diameter of the primary lesion compared with baseline with response evaluation criteria in solid tumors version 1.1 (RECIST v1.1) measurement. **(B)** The duration of response in patients undergoing PD-1 inhibitor and chemotherapy.

At the follow-up deadline of June 30, 2024, 3 patients (3.8%) in the TPF-ICB group experienced disease progression, significantly lower than 12 patients (15%) in the TPF group (*p* = 0.016) (**Figures 1**, **2**). The addition of PD-1 inhibitor can reduce the disease recurrence of LA-NPC patients by 74.67%. Additionally, the number of deaths in the TPF-ICB group (3 patients, 3.8%) was significantly lower than that in the TPF group (9 patients, 11.2%, *p* < 0.0001) (**Figures 1**, **2**).

### Toxicity assessment

In the TPF group, common adverse events included alopecia, peripheral nerve numbness, vomiting, decreased appetite, leukopenia, and neutropenia. Grade 1-2 adverse events occurred in all patients, and grade 3-4 adverse reactions occurred in a total of 7 cases (8.6%). In the TPF-ICB group, common adverse events included alopecia, peripheral nerve numbness, neutropenia, hypoalbuminemia, leukopenia, decreased appetite, and vomiting. Grade 1-2 adverse events occurred in all patients, and grade 3-4 adverse events occurred in a total of 12 cases (15.5%). There was no statistical difference in adverse effects between the two groups. There were no treatment-related deaths in either group (**Table 3**).

**Table 3:** Treatment related adverse events in induction chemotherapy combined with or without PD-1 inhibitor.

Variables	TPF		TPF-ICB		*P*
	Grade 1-2	Grade 3-4	Grade 1-2	Grade 3-4	
Hypokalemia	2(2.5%)	0	6(7.7%)	0	
Neutropenia	11(13.7%)	2(2.4%)	14(17.9%	6(7.8%)	0.424
Leukopenia	12(15.0%)	4(5.0%)	13(16.6%)	5(6.4%)	0.949
Thrombocytopenia	6(7.6%)	1(1.2%)	4(5.1%)	1(1.3%)	0.562
Hypoalbuminemia	5(6.2%)	0	13(16.7%)	0	0.096
Alanine aminotransferase	0	0	1(1.3%)	0	0.31
Elevated creatinine	2(1.9%)	0	1(1.3%)	0	0.575
Decreased appetite	13(16.2%)	0	13(16.6%)	0	0.327
Nausea	4(5.0%)	0	9(11.6%)	0	0.266
Vomiting	14(17.4%)	0	10(12.8%)	0	0.694
Weight loss	6(7.5%)	0	1(1.3%)	0	0.182
Fatigue	5(6.2%)	0	4(5.1%)	0	0.761
Abnormal peripheral sensory nerves	19(23.8%)	0	22(28.3%)	0	0.733
Alopecia	78(100%)	0	78(100%)	0	0.357
Hypothyroidism	2(3.1%)	0	3(3.9%)	0	0.596
Allergic reactions	0	0	0	0	-
Vertigo	0	0	0	0	-
Venous thrombosis	0	0	0	0	-
Elevated total bilirubin	0	0	0	0	-
Indirect elevation of bilirubin	0	0	0	0	-
Direct elevation of bilirubin	0	0	0	0	-
Cough	0	0	0	0	-
Aspartate aminotransferase	0	0	0	0	-
Diarrhea	0	0	0	0	-

ICB, PD-1 inhibitor immunotherapy; TPF, nab-paclitaxel, cisplatin, and 5-fluorouracil.

### PFS and OS

**Figure 3:**
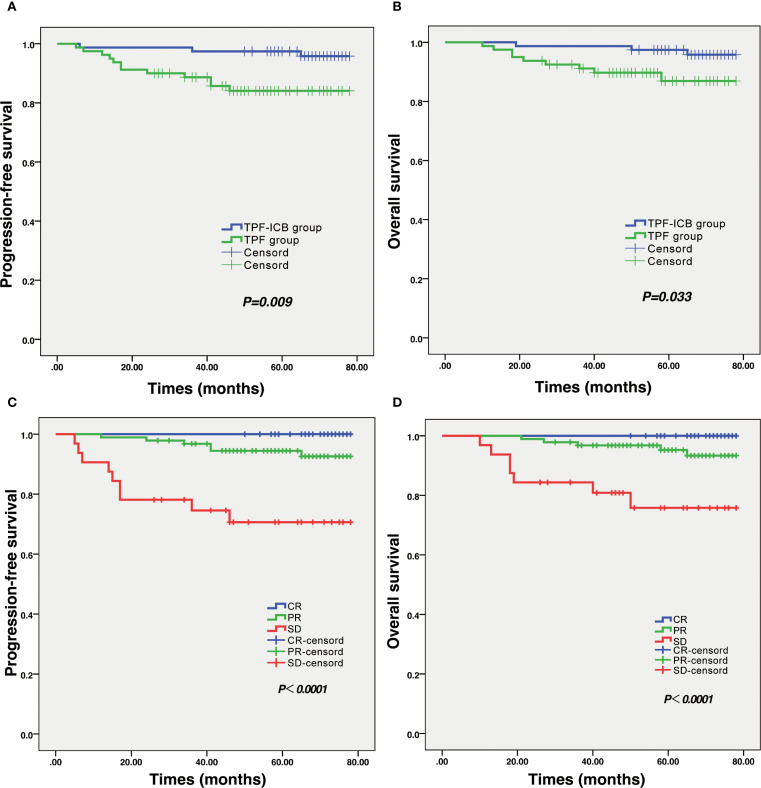
Results of survival analysis and efficacy evaluation in LA-NPC patients. **(A)** Survival curve of PFS in TPF and TPF-ICB groups. **(B)** Survival curve of OS in TPF and TPF-ICB groups. **(C)** PFS survival curves between CR, PR, and SD groups after induction therapy. **(D)** OS survival curves between CR, PR, and SD groups after induction therapy. CR, complete response; ICB, PD-1 inhibitor immunotherapy; OS, overall survival; PFS, progressive free survival; PR, partial response; SD, stable disease; TPF, nab-paclitaxel, cisplatin, and 5-fluorouracil.

The mean ± standard deviation (SD) of PFS time in the TPF group was 51.36 ± 19.29 months, significantly lower than the 67.51 ± 10.80 months in the TPF-ICB group (*p* = 0.009) (**Figure 3A**). The mean ± SD of OS time in the TPF group was 52.90 ± 17.46 months, also significantly lower than the 67.86 ± 9.45 months in the TPF-ICB group (*p* = 0.033) (**Figure 3B**).

In addition, this study also found that tumor efficacy evaluation results after induction therapy significantly affected patients’ survival time. Kaplan-Meier survival curve plots showed that PFS and OS in CR patients were significantly higher than those in PR and SD patients after induction therapy. There were significant differences in PFS (*p* < 0.001) and OS (*p* < 0.001) among the CR, PR, and SD groups (**Figures 3C, D**). In addition, there were no statistically significant differences in PFS (*p* = 0.933) and OS (*p* = 0.483) among patients in different immunotherapy regimens, regardless of whether they were combined with toripalimab, sintilimab, or tislelizumab during induction chemotherapy.

### Prognostic factors

In order to investigate predictive factors that influence patient prognosis, we performed univariate and multivariate analyses on a total of 158 patients. As shown in **Table 4**, the univariate analysis demonstrated that disease stage (*p* = 0.033), the efficacy of induction therapy (*p* < 0.0001) and induced immunotherapy (*p =* 0.009) were prognostic factors for PFS. However, the results of the multivariate analysis revealed that only the efficacy of induction therapy was an independent factor for PFS (*p* = 0.001, HR 5.938, 95% CI 2.064-17.084) (**Table 4**).

**Table 4:** Univariate and multivariate analysis of prognostic factors for overall survival.

Variables	Univariate analysis	Cox multivariate analysis	
		HR [95%CI]	*P*
Age (>50 vs. ≤50)	0.543		
Sex (Male vs. female)	0.155		
Smoker (Yes vs. No)	0.372		
Drinker (Yes vs. No)	0.223		
Ki67 (>30% vs. ≤30%)	0.486		
ECOG (1 vs. 0)	0.209		
Disease stage (III vs. IVA)	0.2		
Tumor stage (T4 vs. T1 +T2 +T3)	0.665		
Neck stage (N+ vs. N0)	0.589		
BMI (≥24 vs.18.5-24 vs. ≤18.5)	0.125		
The efficacy of induction therapy (CR vs. PR vs. SD)	0.001	5.324 [1.556-18.215]	0.008
Induction immunotherapy	0.033		

CR, complete response; ECOG, Eastern Cooperative Oncology Group; PR, partial response; SD, stable disease.

The univariate analysis indicated that the efficacy of induction therapy (*p* = 0.001) and induced immunotherapy (*p* = 0.033) were independent risk factors for OS. Similarly, the results of multivariate analysis showed that only the efficacy of induction therapy (*p* = 0.008, HR 5.324, 95% CI 1.556-18.215) were independent prognostic factor for OS (**Table 5**).

**Table 5:** Univariate and multivariate analysis of prognostic factors for progression free survival.

Variables	Univariate analysis	Cox multivariate analysis	
		HR [95%CI]	*P*
Age (>50 vs. ≤50)	0.321		
Sex (Male vs. female)	0.438		
Smoker (Yes vs. No)	0.735		
Drinker (Yes vs. No)	0.851		
Ki67 (>30% vs. ≤30%)	0.508		
ECOG (1 vs. 0)	0.188		
Disease stage (III vs. IVA)	0.033		
Tumor stage (T4 vs. T1 +T2 +T3)	0.474		
Neck stage (N+ vs. N0)	0.53		
BMI (≥24 vs.18.5-24 vs. ≤18.5)	0.205		
The efficacy of induction therapy (CR vs. PR vs. SD)	<0.0001	5.938 [2.064-17.084]	0.001
Induction immunotherapy	0.009		

CR, complete response; ECOG, Eastern Cooperative Oncology Group; PR, partial response; SD, stable disease.

## Discussion

This retrospective study major explored the safety and efficacy of TPF induced chemotherapy combined with PD-1 inhibitor in LA-NPC patients. The most important finding in current research is that the addition of PD-1 inhibitors to LA-NPC induced chemotherapy does not significantly increase the risk of adverse events in patients. On the contrary, it is more beneficial for tumor remission during induction therapy.

Induction chemotherapy based on gemcitabine + cisplatin (GP) or docetaxel+ cisplatin + fluorouracil combined with CCRT is one of the standard treatment methods for LA-NPC (17, 29, 36, 37). Despite undergoing induction chemotherapy in conjunction with CCRT, 20-30% of LA-NPC patients still face recurrence of the disease (16, 17, 38). Therefore, it is necessary to seek new treatment methods to improve the OS of this population. Due to the abundant lymphocyte infiltration in the microenvironment of NPC, which is even referred to as “lymphoepithelioma”, it may be one of the effective mechanisms for PD-1/PD-L1 therapy (20, 23, 26).

The PD-1/PD-L1 signaling pathway is an important pathway in the mechanism of tumor escape (39). PD-L1 binds to PD-1 through a series of signaling pathways, ultimately inhibiting the transcription and translation of genes and cytokines required for T cell activation, exerting a negative regulatory effect on T cell activity, leading to tumor immune escape and promoting tumor growth (40). The anti-PD-1/PD-L1 monoclonal antibody can block the binding of PD-L1 on the surface of tumor cells to T cell PD-1, relieve the suppression of tumor cell immune function, activate immune function, and thus kill tumor cells (41, 42). Immunotherapy is different from traditional therapies in that it enhances efficacy by dynamically regulating the tumor microenvironment and anti-tumor immunity, and can be combined with other therapies to further improve the immune microenvironment and enhance treatment efficacy (43). The rationale for these combination strategies is multifactorial, involving synergistic mechanisms such as (1) enhanced T-cell activation through complementary immune checkpoint blockade (CTLA-4 inhibition promotes T-cell priming, while PD-1/PD-L1 blockade reverses effector T-cell exhaustion in peripheral tissues); (2) increased tumor immunogenicity via elevated antigen exposure, neoantigen presentation, and tumor mutational burden; (3) upregulation of PD-L1 expression to potentiate checkpoint inhibition; and (4) improved T-cell trafficking to metastatic sites with consequent remodeling of the tumor microenvironment toward an immune-permissive state (44, 45). This multimodal approach capitalizes on distinct yet complementary pathways to overcome tumor immune evasion more effectively than monotherapies.

Currently, PD-1 inhibitor combined chemotherapy has been approved as the first-line standard treatment for R/M-NPC. Similar to classical Hodgkin’s lymphoma, NPC features a unique lymphocyte infiltration environment, making it more susceptible to the benefits of PD-1 inhibitors (46–48). The effectiveness and safety of many PD-1 inhibitors have been demonstrated in patients with R/M-NPC. A phase I clinical trial of using camrelizumab to treat R/M-NPC showed that 34% (95% CI: 24%-44%) of patients achieved disease remission during a median follow-up period of 9.9 months (22). A phase III clinical trial found that compared with GP regimen chemotherapy, the combination of camrelizumab and GP regimen can significantly prolong the PFS of R/M-NPC patients (9.7 months vs. 6.9 months, *p* = 0.0002), and does not significantly increase the toxicity and side effects of patients (23). Similarly, another multicenter randomized phase III clinical study confirmed that the combination of toripalimab and GP regimen chemotherapy significantly improved PFS in R/M-NPC patients (11.7 months vs. 8.0 months, HR = 0.52 (95% CI: 0.36-0.74), *p* = 0.0003) (21). The results of RATIONALE 309 further confirm that tislelizumab combined with GP regimen chemotherapy can become the first-line standard treatment for R/M-NPC patients (20). Therefore, based on the results of the three studies mentioned above, PD-1 inhibitor with GP regimen has been approved as the first-line standard treatment for R/M-NPC.

Based on preclinical evidence and promising results of PD-1 inhibitors in RM-NPC, adding immunotherapy to induction therapy for LA-NPC patients is a popular clinical research topic. Research has shown that after induction chemotherapy, the bone marrow-derived suppressor cells, regulatory T cells, and B cells in the peripheral blood of NPC patients are significantly reduced, while CD3 T cells, central memory T cells, and pro-inflammatory cytokines are significantly increased. This provides a solid foundation for exploring the combination of immunotherapy and induction chemotherapy for NPC (49).

A retrospective study included 57 LA-NPC patients who received induction chemotherapy combined with anti-PD-1 immunotherapy, which significantly increased the ORR rate of primary nasopharynx from 68.60% in the induction chemotherapy group (121 patients) alone to 94.7% (50). Another retrospective study included 217 LA-NPC patients, of whom 67 received induction chemotherapy combined with anti-PD-1 treatment and 150 received induction chemotherapy alone. The results showed that the ORR of the induction chemotherapy combined with anti-PD-1 group was 88.1% (59/67), while the induction chemotherapy group was 70.0% (105/150) (51). Similarly, another retrospective study found that the ORR of neoadjuvant therapy with Tislelizumab combined with nab-paclitaxel and cisplatin chemotherapy in 43 patients with LA-NPC was 88.4%, significantly higher than the 70.2% in the induction chemotherapy group alone, and the 3-year PFS rate was also significantly improved (93.0% vs. 78.7%) (52). Although their research results are similar to ours, their sample size is smaller and the follow-up time is shorter. The ORR results are consistent with our study (88.5%). In addition, a phase II clinical study found that compared with 50 LA-NPC patients receiving simple CCRT treatment, 100 patients who received two cycles of induction therapy with toripalimab, followed by CCRT, and then received toripalimab maintenance therapy, known as the “immune sandwich” treatment model, significantly improved their 2-year PFS rate (92.0% vs. 74.0%), and reduced the risk of disease progression or death by 60% (53). But, our TPF-based induction regimen combined with PD-1 blockade demonstrated even higher 2-year PFS or OS rates (99.0%). The main reason may be that Mai et al.’ study only used 2 cycles of toripalimab immunotherapy in induction therapy, without combination chemotherapy, and had a shorter median follow-up time (37.8 months) (53).

A randomized controlled phase 3 clinical study on the combination of GP induced chemotherapy and CCRT therapy with sintilimab for the treatment of 210 LA-NPC also showed that adding PD-1 inhibitors to standard treatment increased the 3-year event-free survival rate from 76% to 86% (median follow-up was 41·9 months) (27). However, our study found that the 3-year survival rate was higher after TPF combined with PD-1 monoclonal antibody induction therapy, which may be due to the significantly lower proportion of IVA patients included and longer median follow-up time (65 months) compared to Ma et al.’s study (41.8% vs. 70%) (27). In addition, for patients initially diagnosed with LA-NPC, there are still a number of phase III clinical trials of adding PD-1 inhibitors to induction chemotherapy and CCRT are currently in the participant recruitment stage (NCT04453826, NCT04557020) (54). This represents one of the larger reported cohorts evaluating PD-1 inhibitors combined with TPF induction chemotherapy for LA-NPC. Although the sample size in this study is smaller than Ma Jun’s clinical research (27), it is comparable to or even exceeds the sample size of several other key trials on NPC induced chemotherapy (50, 51, 53).

To our knowledge, this is the first real-world study evaluating TPF induction chemotherapy combined with PD-1 inhibitors for LA-NPC. Our study provides robust evidence supporting the incorporation of PD-1 inhibitors into TPF induction chemotherapy for LA-NPC, with a median follow-up of 65 months—one of the longest reported in this setting. Unlike previous studies (27, 49, 51, 53), our TPF-ICB approach leveraged the nab-paclitaxel can enhance the efficacy of immunotherapy by regulating the cancer cell cycle, which may explain the superior ORR and survival outcomes (55). Our findings align with recent studies supporting the integration of immunotherapy in NPC. The CAPTAIN-NPC trial (NCT03707509) reported improved PFS with camrelizumab plus chemotherapy in recurrent/metastatic NPC (23), while the JUPITER-02 study (NCT03581786) demonstrated survival benefits with toripalimab in combination with gemcitabine/cisplatin (21). Although these trials focused on metastatic disease, our data extend these observations to the LA-NPC setting, reinforcing the potential of PD-1 inhibitors in curative-intent treatment. Mechanistically, PD-1 blockade may counteract immune evasion by EBV-associated NPC, which typically exhibits high PD-L1 expression and tumor-infiltrating lymphocytes (3).

The superior ORR and survival in our TPF-ICB group may also reflect synergistic effects between chemotherapy and immunotherapy. Preclinical evidence suggests that chemotherapy enhances antigen presentation and T-cell activation, potentially augmenting PD-1 inhibitor efficacy (56). Many chemotherapeutic agents, including cisplatin, 5-fluorouracil, and nab-paclitaxel, can upregulate multiple surface molecules on tumor cells, rendering them more sensitive to immune-mediated killing (57–59). TPF regimens may reduce immunosuppressive cells [e.g., myeloid-derived suppressor cells (MDSCs), regulatory T cells (Tregs)] while increasing CD8 T-cell infiltration, synergizing with PD-1 blockade (60–62). In EBV-associated tumors, chemotherapy may amplify viral antigen exposure, enhancing PD-1 inhibitor-driven immune responses (63–65). Additionally, our cohort included three PD-1 inhibitors (sintilimab, tislelizumab, toripalimab), all showing comparable activity—a finding consistent with the CLASSIC study (NCT04398056), which reported no significant efficacy differences among anti-PD-1 agents in NPC (66).

On the other hand, we analyzed the safety of PD-1 inhibitor combined with chemotherapy in the induction therapy of LA-NPC. In our real-world study, the most common adverse events in patients during immunotherapy combined with TPF-induced chemotherapy included hair loss (100%), peripheral nerve numbness (23.8%), vomiting (17.4%) and decreased appetite (16.2%). In addition, the incidence of adverse events in PD-1 inhibitor combined with TPF group was not significantly higher than that in TPF chemotherapy group alone. Overall, the incidence of adverse events, with the exception of hair loss, was lower than in previous studies (67). This also indicates that the use of PD-1 inhibitors combined with TPF chemotherapy in the induction therapy of LA-NPC is beneficial for improving tumor shrinkage rate, prolonging patient survival time, and has controllable safety. While our median 65-month follow-up adequately captures acute and subacute toxicities, longer observation is needed to fully evaluate late-onset immune-related effects and cumulative treatment toxicities. This represents an important direction for continued monitoring of this cohort. In addition, this study found that both the 2-year PFS and OS rates of patients after induction therapy with PD-1 inhibitor combined with TPF regimen were 99.0%, and the ORR was 88.5%, which were higher than previous studies (67). We found that the addition of PD-1 inhibitors in induction chemotherapy further improved the 3-year and 5-year PFS and OS rates of LA-NPC patients. This study also found that the addition of PD-1 inhibitors can not only improve the tumor remission rate of LA-NPC patients after induction therapy, but also significantly reduce the risk of recurrence in patients.

However, the discussion on the optimal induction therapy for LA-NPC is still ongoing, and the optimal sequence of PD-1 inhibitors (induction and maintenance) and long-term outcomes (over 5 years) are still unclear. In any case, we have seen a glimmer of hope in PD-1 monoclonal immunotherapy in LA-NPC patients, and hope to be confirmed by more study results and longer follow-up. Although the heterogeneity of this study is relatively high, considering that we are attempting to explore the efficacy of PD-1/PD-L1 inhibitors and their impact on NPC from a holistic perspective, this is acceptable. On the one hand, compared with standard treatment, the combination chemotherapy of sintilimab, tislelizumab, and toripalimab has shown encouraging therapeutic effects and good tolerability in NPC patients. On the other hand, there were no statistically significant differences in PFS and OS among patients in different immunotherapy regimens. Moving forward, we highlight three critical research directions: biomarker discovery to identify optimal candidates, optimization of treatment sequencing to maximize synergy while minimizing toxicity, and comprehensive tumor microenvironment characterization using single-cell technologies to elucidate the dynamic immune changes induced by this combination therapy (54, 65, 68, 69). These additions strengthen the biological rationale for our clinical findings while outlining a translational roadmap for future investigation.

Despite these promising results, our study has limitations. Firstly, its retrospective design may introduce selection bias, though baseline characteristics were balanced (*p* > 0.05). Some other concomitant diseases, such as diabetes and hypertension, may affect the prognosis of patients with NPC (70). However, due to the limitations of retrospective studies, this article has not included an analysis of comorbidities. Secondly, due to the accessibility of data, many hematological test indicators, including hemoglobin, platelets, white blood cells, neutrophils, EBV DNA copy number, etc., were not included in the analysis, although these indicators are considered prognostic markers for NPC patients in many studies (71–75). Thirdly, our sample size is relatively small. Overall, our findings demonstrate that these inhibitors maintain comparable real-world efficacy to clinical trial results for LA-NPC. Future randomized prospective studies should address these gaps and exploring predictive biomarkers (e.g., PD-L1, tumor mutational burden and hematological test indicators) to evaluate their potential prognostic value in LA-NPC combination therapy based on PD-1 inhibitors.

## Conclusion

This study demonstrates that PD-1 inhibitors combined with TPF induction chemotherapy yield exceptional short-term efficacy and manageable toxicity in LA-NPC, expanding upon prior GP-based immunotherapy trials. The regimen’s innovation lies in its synergy potential and applicability to high-risk subgroups (T4/N3). Larger randomized studies with extended follow-up are warranted to validate these findings and refine patient selection criteria.

## Data Availability

The raw data supporting the conclusions of this article will be made available by the authors, without undue reservation.
